# 1-(3-Hy­droxy-5,8-dimeth­oxy-4-methyl-1,2,3,4-tetra­hydro-1,4-ep­oxy­naphthalen-2-yl)ethan-1-one

**DOI:** 10.1107/S1600536814007764

**Published:** 2014-04-12

**Authors:** Alan J. Lough, Jaipal R. Nagireddy, William Tam

**Affiliations:** a Department of Chemistry, University of Toronto, Toronto, Ontario, M5S 3H6, Canada; b Department of Chemistry, University of Guelph, Guelph, Ontario, N1G 2W1, Canada

## Abstract

The stereochemistry and regioschemistry (*exo*) of the title compound, C_15_H_18_O_5_, were determined by the X-ray analysis. The meth­oxy groups essentially lie in the plane of the benzene ring to which they are attached, as described by the C—O—C C torsion angles of −176.80 (12) and 4.67 (19)°. In the crystal, O—H⋯O hydrogen bonds and weak C—H⋯O hydrogen bonds link the mol­ecules, forming chains of *R*_2_^1^(8) rings along [010].

## Related literature   

For the metal-mediated cleavage of 2-isoxazoline rings fused to bicyclic frameworks, see: Tranmer & Tam (2002 ▶). For hydrogen-bond graph-set notation, see: Bernstein *et al.* (1995 ▶).
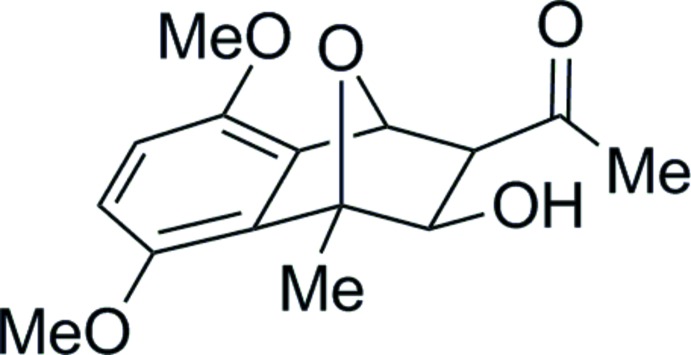


## Experimental   

### 

#### Crystal data  

C_15_H_18_O_5_
*M* *_r_* = 278.29
Orthorhombic, 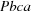

*a* = 10.3091 (4) Å
*b* = 9.1309 (4) Å
*c* = 29.2552 (13) Å
*V* = 2753.8 (2) Å^3^
*Z* = 8
Cu *K*α radiation
μ = 0.84 mm^−1^
*T* = 147 K
0.17 × 0.14 × 0.05 mm

#### Data collection  

Bruker Kappa APEX DUO CCD diffractometer
Absorption correction: multi-scan (*SADABS*; Bruker, 2012 ▶) *T*_min_ = 0.686, *T*_max_ = 0.753
9265 measured reflections
2368 independent reflections
2195 reflections with *I* > 2σ(*I*)
*R*_int_ = 0.021

#### Refinement  

*R*[*F* ^2^ > 2σ(*F* ^2^)] = 0.036
*wR*(*F* ^2^) = 0.097
*S* = 1.04
2368 reflections
189 parameters
H atoms treated by a mixture of independent and constrained refinement
Δρ_max_ = 0.42 e Å^−3^
Δρ_min_ = −0.24 e Å^−3^

### 

Data collection: *APEX2* (Bruker, 2012 ▶); cell refinement: *SAINT* (Bruker, 2012 ▶); data reduction: *SAINT*; program(s) used to solve structure: *SHELXS97* (Sheldrick, 2008 ▶); program(s) used to refine structure: *SHELXL2013* (Sheldrick, 2008 ▶); molecular graphics: *PLATON* (Spek, 2009 ▶); software used to prepare material for publication: *SHELXTL* (Sheldrick, 2008 ▶).

## Supplementary Material

Crystal structure: contains datablock(s) I. DOI: 10.1107/S1600536814007764/tk5301sup1.cif

Structure factors: contains datablock(s) I. DOI: 10.1107/S1600536814007764/tk5301Isup2.hkl

Supporting information file. DOI: 10.1107/S1600536814007764/tk5301Isup3.cml

CCDC reference: 995954

Additional supporting information:  crystallographic information; 3D view; checkCIF report

## Figures and Tables

**Table 1:** Hydrogen-bond geometry (Å, °)

*D*—H⋯*A*	*D*—H	H⋯*A*	*D*⋯*A*	*D*—H⋯*A*
O2—H2*O*⋯O3^i^	0.97 (3)	1.89 (2)	2.8295 (15)	162 (2)
C7—H7*C*⋯O3^i^	0.98	2.42	3.3936 (19)	173

Symmetry code: (i) 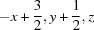.

**Fig. 1.**
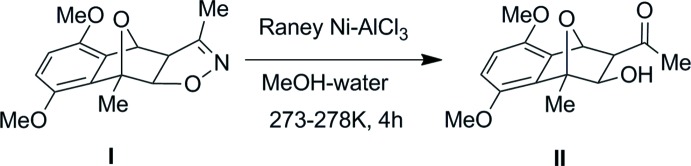
The reaction scheme.

**Fig. 2.**
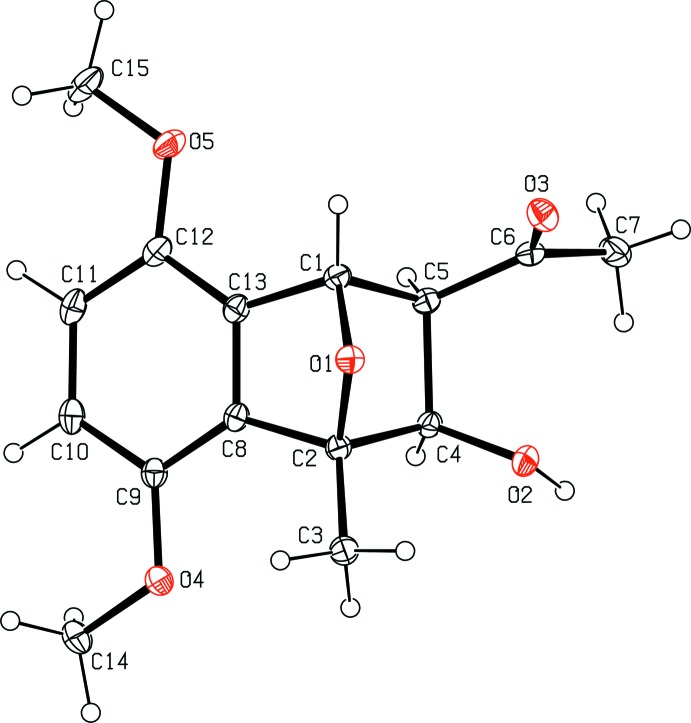
The molecular structure of the title compound showing 30% probability ellipsoids.

**Fig. 3.**
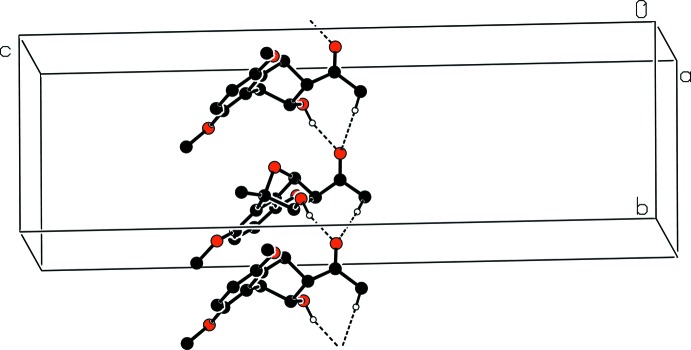
Part of the crystal structure with weak hydrogen bonds shown as dashed lines.

## supplementary crystallographic information

### 2. Structural commentary

We have previously investigated the metal-mediated cleavage of 2-isoxazoline rings fused to bicyclic frameworks that are prepared in our laboratory through nitrile oxide 1,3-dipolar cyclo­addition methodology (Tranmer & Tam, 2002). When expanding this study using Raney Ni, cleavage of 2-isoxazoline compound (I) (see Fig. 1) leads to the formation of β-hy­droxy ketone product (II) as a single stereoisomer. The stereochemistry of the product was determined by this single-crystal X-ray analysis. Although different stereoisomers (*exo* and *endo*) could be formed, only the *exo* regioisomer (II) was formed.

The molecular structure of the title compound is shown in Fig. 2. The meth­oxy groups essentially lie in the plane of the benzene ring to which they are attached as described by the C15—O5—C12—C11 and C14—O4—C9—C10 torsion angles of -176.80 (12) and 4.67 (19)°. In the crystal, O—H···O hydrogen bonds and weak C—H···O hydrogen bonds link molecules forming chains of *R*^1^_2_(8) rings (Bernstein *et al.*, 1995) along [010] (see Fig. 3).

### 5. Synthesis and crystallization

To an oven dried flask containing the cyclo­adduct (I) (60 mg, 0.21 mmol) was added methanol (5 ml), THF (5 ml) and distilled water (2 ml) and the mixture was cooled to 273–278 K using an ice bath. AlCl_3_ (87 mg, 0.65 mmol) was added to the cold solution in one portion and was stirred at 273 K for 15 minutes. Raney-nickel (0.52 g) was added and was stirred for 4 h at 273 K. The reaction mixture was filtered through a pad of celite, while washing with di­chloro­methane (20 ml). The organic layer was separated and the aqueous layer was extracted again with di­chloro­methane (10 ml), and the combined organic layers were dried over sodium sulfate then evaporated using rotary evaporation. The crude product was washed by hexanes (3 ml) followed by recrystallizaion in EtOAc:hexanes = 1:5 to give product (II) in 86% yield. X-ray quality crystals were grown from a solution of the title compound in EtOAc:hexanes = 1:5.

### 6. Refinement

Hydrogen atoms bonded to C atoms were placed in calculated positions with C—H distances ranging from 0.95–1.00 Å and included in the refinement in a riding-model approximation with *U*_iso_(H) = 1.2*U*_eq_(C) or 1.5*U*_eq_(C_methyl_). The hydroxyl H atom was refined independently with an isotropic displacement parameter.

### Crystal data

**Table:**

C_15_H_18_O_5_	*D*_x_ = 1.342 Mg m^−^^3^
*M**_r_* = 278.29	Cu *K*α radiation, λ = 1.54178 Å
Orthorhombic, *P**b**c**a*	Cell parameters from 6136 reflections
*a* = 10.3091 (4) Å	θ = 5.3–66.6°
*b* = 9.1309 (4) Å	µ = 0.84 mm^−^^1^
*c* = 29.2552 (13) Å	*T* = 147 K
*V* = 2753.8 (2) Å^3^	Plate, colourless
*Z* = 8	0.17 × 0.14 × 0.05 mm
*F*(000) = 1184	

### Data collection

**Table:**

Bruker Kappa APEX DUO CCD diffractometer	2195 reflections with *I* > 2σ(*I*)
Radiation source: Bruker ImuS with multi-layer optics	*R*_int_ = 0.021
φ and ω scans	θ_max_ = 66.6°, θ_min_ = 5.3°
Absorption correction: multi-scan (*SADABS*; Bruker, 2012)	*h* = −11→10
*T*_min_ = 0.686, *T*_max_ = 0.753	*k* = −6→10
9265 measured reflections	*l* = −34→34
2368 independent reflections	

### Refinement

**Table:**

Refinement on *F*^2^	0 restraints
Least-squares matrix: full	Hydrogen site location: mixed
*R*[*F*^2^ > 2σ(*F*^2^)] = 0.036	H atoms treated by a mixture of independent and constrained refinement
*wR*(*F*^2^) = 0.097	*w* = 1/[σ^2^(*F*_o_^2^) + (0.0532*P*)^2^ + 1.2534*P*] where *P* = (*F*_o_^2^ + 2*F*_c_^2^)/3
*S* = 1.04	(Δ/σ)_max_ = 0.001
2368 reflections	Δρ_max_ = 0.42 e Å^−^^3^
189 parameters	Δρ_min_ = −0.24 e Å^−^^3^

### Special details

**Table:**

Geometry. All e.s.d.'s (except the e.s.d. in the dihedral angle between two l.s. planes) are estimated using the full covariance matrix. The cell e.s.d.'s are taken into account individually in the estimation of e.s.d.'s in distances, angles and torsion angles; correlations between e.s.d.'s in cell parameters are only used when they are defined by crystal symmetry. An approximate (isotropic) treatment of cell e.s.d.'s is used for estimating e.s.d.'s involving l.s. planes.

### Fractional atomic coordinates and isotropic or equivalent isotropic displacement parameters (Å^2^)

**Table:**

	*x*	*y*	*z*	*U*_iso_*/*U*_eq_	
O1	0.88037 (9)	0.52360 (10)	0.62898 (3)	0.0212 (2)	
O2	0.70291 (9)	0.72212 (11)	0.58154 (3)	0.0259 (2)	
O3	0.85382 (10)	0.46468 (11)	0.52476 (3)	0.0294 (3)	
O4	0.93569 (9)	0.88101 (11)	0.72203 (3)	0.0267 (2)	
O5	1.28252 (9)	0.59024 (12)	0.60787 (3)	0.0315 (3)	
C1	0.99163 (13)	0.55891 (15)	0.60115 (4)	0.0212 (3)	
H1A	1.0361	0.4727	0.5871	0.025*	
C2	0.85197 (12)	0.67220 (14)	0.64418 (4)	0.0192 (3)	
C3	0.73874 (13)	0.67520 (16)	0.67653 (4)	0.0250 (3)	
H3A	0.7645	0.6323	0.7059	0.038*	
H3B	0.6669	0.6185	0.6636	0.038*	
H3C	0.7109	0.7767	0.6813	0.038*	
C4	0.83079 (12)	0.74951 (14)	0.59729 (4)	0.0194 (3)	
H4A	0.8488	0.8569	0.5994	0.023*	
C5	0.93447 (13)	0.66931 (14)	0.56674 (4)	0.0204 (3)	
H5A	1.0027	0.7399	0.5566	0.025*	
C6	0.87214 (13)	0.59612 (15)	0.52575 (4)	0.0230 (3)	
C7	0.84055 (16)	0.69186 (18)	0.48585 (5)	0.0328 (4)	
H7A	0.7895	0.6362	0.4636	0.049*	
H7B	0.9211	0.7256	0.4715	0.049*	
H7C	0.7903	0.7766	0.4963	0.049*	
C8	0.98548 (13)	0.71876 (14)	0.66166 (4)	0.0192 (3)	
C9	1.02832 (13)	0.81205 (14)	0.69594 (4)	0.0212 (3)	
C10	1.16170 (14)	0.82634 (16)	0.70211 (5)	0.0259 (3)	
H10A	1.1935	0.8868	0.7261	0.031*	
C11	1.24964 (14)	0.75349 (16)	0.67375 (5)	0.0276 (3)	
H11A	1.3401	0.7656	0.6786	0.033*	
C12	1.20648 (13)	0.66370 (15)	0.63864 (5)	0.0242 (3)	
C13	1.07345 (13)	0.64562 (15)	0.63424 (4)	0.0213 (3)	
C14	0.98079 (15)	0.98213 (16)	0.75556 (5)	0.0299 (3)	
H14A	0.9064	1.0277	0.7708	0.045*	
H14B	1.0333	1.0580	0.7407	0.045*	
H14C	1.0337	0.9302	0.7782	0.045*	
C15	1.41953 (14)	0.60214 (19)	0.61366 (6)	0.0354 (4)	
H15A	1.4636	0.5488	0.5891	0.053*	
H15B	1.4443	0.5603	0.6433	0.053*	
H15C	1.4448	0.7055	0.6126	0.053*	
H2O	0.673 (2)	0.813 (3)	0.5676 (8)	0.072 (7)*	

### Atomic displacement parameters (Å^2^)

**Table:**

	*U*^11^	*U*^22^	*U*^33^	*U*^12^	*U*^13^	*U*^23^
O1	0.0214 (5)	0.0188 (5)	0.0234 (5)	−0.0021 (4)	0.0009 (4)	−0.0006 (4)
O2	0.0188 (5)	0.0323 (6)	0.0267 (5)	0.0002 (4)	−0.0033 (4)	−0.0005 (4)
O3	0.0325 (6)	0.0294 (6)	0.0263 (5)	−0.0037 (4)	−0.0013 (4)	−0.0039 (4)
O4	0.0249 (5)	0.0278 (5)	0.0274 (5)	0.0002 (4)	−0.0040 (4)	−0.0091 (4)
O5	0.0177 (5)	0.0428 (6)	0.0341 (6)	0.0056 (4)	0.0026 (4)	0.0006 (5)
C1	0.0181 (7)	0.0226 (7)	0.0227 (7)	0.0012 (5)	0.0010 (5)	−0.0012 (5)
C2	0.0179 (7)	0.0183 (7)	0.0214 (6)	−0.0010 (5)	−0.0006 (5)	−0.0021 (5)
C3	0.0199 (7)	0.0322 (8)	0.0229 (7)	−0.0026 (6)	0.0019 (5)	−0.0003 (6)
C4	0.0155 (6)	0.0210 (7)	0.0217 (7)	−0.0008 (5)	−0.0009 (5)	−0.0013 (5)
C5	0.0177 (7)	0.0215 (7)	0.0221 (7)	0.0000 (5)	0.0016 (5)	0.0001 (5)
C6	0.0185 (7)	0.0280 (8)	0.0225 (7)	0.0030 (5)	0.0035 (5)	−0.0016 (6)
C7	0.0397 (9)	0.0377 (9)	0.0211 (7)	0.0107 (7)	−0.0001 (6)	−0.0010 (6)
C8	0.0183 (7)	0.0187 (6)	0.0208 (6)	−0.0008 (5)	−0.0017 (5)	0.0034 (5)
C9	0.0224 (7)	0.0198 (6)	0.0214 (6)	−0.0005 (5)	−0.0027 (5)	0.0021 (5)
C10	0.0250 (8)	0.0255 (7)	0.0271 (7)	−0.0057 (6)	−0.0075 (6)	0.0017 (6)
C11	0.0185 (7)	0.0324 (8)	0.0319 (7)	−0.0034 (6)	−0.0059 (6)	0.0063 (6)
C12	0.0184 (7)	0.0270 (7)	0.0273 (7)	0.0020 (6)	0.0004 (5)	0.0062 (6)
C13	0.0199 (7)	0.0215 (7)	0.0223 (7)	0.0008 (5)	−0.0013 (5)	0.0033 (5)
C14	0.0334 (8)	0.0292 (7)	0.0270 (7)	−0.0034 (6)	−0.0042 (6)	−0.0093 (6)
C15	0.0171 (8)	0.0468 (10)	0.0422 (9)	0.0059 (7)	0.0030 (6)	0.0146 (7)

### Geometric parameters (Å, º)

**Table:**

O1—C1	1.4431 (16)	C5—C6	1.5157 (18)
O1—C2	1.4576 (16)	C5—H5A	1.0000
O2—C4	1.4187 (16)	C6—C7	1.494 (2)
O2—H2O	0.97 (3)	C7—H7A	0.9800
O3—C6	1.2153 (18)	C7—H7B	0.9800
O4—C9	1.3750 (17)	C7—H7C	0.9800
O4—C14	1.4252 (16)	C8—C13	1.3827 (19)
O5—C12	1.3692 (17)	C8—C9	1.3880 (19)
O5—C15	1.4268 (18)	C9—C10	1.393 (2)
C1—C13	1.5086 (19)	C10—C11	1.397 (2)
C1—C5	1.5417 (18)	C10—H10A	0.9500
C1—H1A	1.0000	C11—C12	1.387 (2)
C2—C3	1.5030 (18)	C11—H11A	0.9500
C2—C8	1.5286 (18)	C12—C13	1.387 (2)
C2—C4	1.5581 (18)	C14—H14A	0.9800
C3—H3A	0.9800	C14—H14B	0.9800
C3—H3B	0.9800	C14—H14C	0.9800
C3—H3C	0.9800	C15—H15A	0.9800
C4—C5	1.5740 (18)	C15—H15B	0.9800
C4—H4A	1.0000	C15—H15C	0.9800
			
C1—O1—C2	97.11 (9)	C6—C7—H7A	109.5
C4—O2—H2O	106.0 (14)	C6—C7—H7B	109.5
C9—O4—C14	116.88 (11)	H7A—C7—H7B	109.5
C12—O5—C15	116.83 (12)	C6—C7—H7C	109.5
O1—C1—C13	101.51 (10)	H7A—C7—H7C	109.5
O1—C1—C5	102.17 (10)	H7B—C7—H7C	109.5
C13—C1—C5	106.84 (11)	C13—C8—C9	120.45 (12)
O1—C1—H1A	114.9	C13—C8—C2	105.20 (11)
C13—C1—H1A	114.9	C9—C8—C2	134.34 (12)
C5—C1—H1A	114.9	O4—C9—C8	117.45 (12)
O1—C2—C3	111.41 (11)	O4—C9—C10	124.81 (12)
O1—C2—C8	100.38 (10)	C8—C9—C10	117.72 (13)
C3—C2—C8	118.92 (11)	C9—C10—C11	121.26 (13)
O1—C2—C4	100.44 (10)	C9—C10—H10A	119.4
C3—C2—C4	115.93 (11)	C11—C10—H10A	119.4
C8—C2—C4	107.14 (10)	C12—C11—C10	120.85 (13)
C2—C3—H3A	109.5	C12—C11—H11A	119.6
C2—C3—H3B	109.5	C10—C11—H11A	119.6
H3A—C3—H3B	109.5	O5—C12—C13	116.53 (12)
C2—C3—H3C	109.5	O5—C12—C11	126.35 (13)
H3A—C3—H3C	109.5	C13—C12—C11	117.12 (13)
H3B—C3—H3C	109.5	C8—C13—C12	122.49 (13)
O2—C4—C2	109.65 (10)	C8—C13—C1	105.01 (11)
O2—C4—C5	111.38 (10)	C12—C13—C1	132.43 (13)
C2—C4—C5	101.19 (10)	O4—C14—H14A	109.5
O2—C4—H4A	111.4	O4—C14—H14B	109.5
C2—C4—H4A	111.4	H14A—C14—H14B	109.5
C5—C4—H4A	111.4	O4—C14—H14C	109.5
C6—C5—C1	112.97 (11)	H14A—C14—H14C	109.5
C6—C5—C4	111.50 (11)	H14B—C14—H14C	109.5
C1—C5—C4	101.12 (10)	O5—C15—H15A	109.5
C6—C5—H5A	110.3	O5—C15—H15B	109.5
C1—C5—H5A	110.3	H15A—C15—H15B	109.5
C4—C5—H5A	110.3	O5—C15—H15C	109.5
O3—C6—C7	121.69 (13)	H15A—C15—H15C	109.5
O3—C6—C5	121.34 (12)	H15B—C15—H15C	109.5
C7—C6—C5	116.90 (12)		
			
C2—O1—C1—C13	52.14 (11)	C3—C2—C8—C9	−27.1 (2)
C2—O1—C1—C5	−58.12 (11)	C4—C2—C8—C9	106.73 (16)
C1—O1—C2—C3	−178.08 (10)	C14—O4—C9—C8	−176.91 (12)
C1—O1—C2—C8	−51.22 (11)	C14—O4—C9—C10	4.67 (19)
C1—O1—C2—C4	58.56 (10)	C13—C8—C9—O4	−179.83 (11)
O1—C2—C4—O2	81.46 (11)	C2—C8—C9—O4	1.0 (2)
C3—C2—C4—O2	−38.70 (15)	C13—C8—C9—C10	−1.29 (19)
C8—C2—C4—O2	−174.15 (10)	C2—C8—C9—C10	179.56 (13)
O1—C2—C4—C5	−36.26 (11)	O4—C9—C10—C11	−179.33 (12)
C3—C2—C4—C5	−156.42 (11)	C8—C9—C10—C11	2.2 (2)
C8—C2—C4—C5	68.13 (12)	C9—C10—C11—C12	−0.4 (2)
O1—C1—C5—C6	−85.45 (12)	C15—O5—C12—C13	−176.80 (12)
C13—C1—C5—C6	168.38 (11)	C15—O5—C12—C11	2.9 (2)
O1—C1—C5—C4	33.82 (12)	C10—C11—C12—O5	177.95 (13)
C13—C1—C5—C4	−72.35 (12)	C10—C11—C12—C13	−2.4 (2)
O2—C4—C5—C6	5.64 (15)	C9—C8—C13—C12	−1.6 (2)
C2—C4—C5—C6	122.10 (11)	C2—C8—C13—C12	177.80 (12)
O2—C4—C5—C1	−114.69 (11)	C9—C8—C13—C1	−178.95 (11)
C2—C4—C5—C1	1.77 (12)	C2—C8—C13—C1	0.42 (13)
C1—C5—C6—O3	10.24 (18)	O5—C12—C13—C8	−176.91 (12)
C4—C5—C6—O3	−102.85 (14)	C11—C12—C13—C8	3.4 (2)
C1—C5—C6—C7	−166.79 (12)	O5—C12—C13—C1	−0.3 (2)
C4—C5—C6—C7	80.12 (15)	C11—C12—C13—C1	179.95 (13)
O1—C2—C8—C13	31.93 (12)	O1—C1—C13—C8	−33.15 (13)
C3—C2—C8—C13	153.61 (12)	C5—C1—C13—C8	73.48 (13)
C4—C2—C8—C13	−72.51 (12)	O1—C1—C13—C12	149.85 (14)
O1—C2—C8—C9	−148.83 (14)	C5—C1—C13—C12	−103.52 (16)

### Hydrogen-bond geometry (Å, º)

**Table:**

*D*—H···*A*	*D*—H	H···*A*	*D*···*A*	*D*—H···*A*
O2—H2*O*···O3^i^	0.97 (3)	1.89 (2)	2.8295 (15)	162 (2)
C7—H7*C*···O3^i^	0.98	2.42	3.3936 (19)	173

Symmetry code: (i) −*x*+3/2, *y*+1/2, *z*.
